# Comparative Analysis of Culture Conditions for the Optimization of Carotenoid Production in Several Strains of the Picoeukaryote *Ostreococcus*

**DOI:** 10.3390/md16030076

**Published:** 2018-02-28

**Authors:** Jean-Baptiste Guyon, Valérie Vergé, Philippe Schatt, Jean-Claude Lozano, Marion Liennard, François-Yves Bouget

**Affiliations:** Observatoire Océanologique, UMR 7621 Laboratoire d’Océanographie Microbienne, Université de Pierre et Marie Curie (Paris 06), Sorbonne Universités, 66650 Banyuls-sur-Mer, France; guyon@obs-banyuls.fr (J.-B.G.); verge@obs-banyuls.fr (V.V.); phs@obs-banyuls.fr (P.S.); jclozano@obs-banyuls.fr (J.-C.L.); marion@liennard.net (M.L.)

**Keywords:** *Ostreococcus*, carotenoids, growth rate, light, temperature, salinity

## Abstract

Microalgae are promising sources for the sustainable production of compounds of interest for biotechnologies. Compared to higher plants, microalgae have a faster growth rate and can be grown in industrial photobioreactors. The microalgae biomass contains specific metabolites of high added value for biotechnology such as lipids, polysaccharides or carotenoid pigments. Studying carotenogenesis is important for deciphering the mechanisms of adaptation to stress tolerance as well as for biotechnological production. In recent years, the picoeukaryote *Ostreococcus* *tauri* has emerged as a model organism thanks to the development of powerful genetic tools. Several strains of *Ostreococcus* isolated from different environments have been characterized with respect to light response or iron requirement. We have compared the carotenoid contents and growth rates of strains of *Ostreococcus* (OTTH595, RCC802 and RCC809) under a wide range of light, salinity and temperature conditions. Carotenoid profiles and productivities varied in a strain-specific and stress-dependent manner. Our results also illustrate that phylogenetically related microalgal strains originating from different ecological niches present specific interests for the production of specific molecules under controlled culture conditions.

## 1. Introduction

Microalgae are known to produce molecules of interest for nutraceutic, cosmetic and pharmaceutical applications, including polyunsaturated fatty acids, vitamins and carotenoids [1]. The success of microalgae-derived production of compounds of interest at industrial scale is due to a number of key factors, the rapid accumulation in biomass being often considered one of the most important. Microalgae are a major source of non-enzymatic antioxidants, among which carotenoids are predominant [2]. Marine microalgae are exposed to dramatic fluctuations in light intensity depending on weather conditions resulting from either reduced solar irradiation or mixing of the water column [3]. Excess light energy damages the photosynthetic machinery, whereas insufficient light supply limits photosynthesis. Carotenoids are keys to the regulation of light input to the photosynthetic apparatus. They serve as accessory pigments to increase the capture of photons that are transferred to chlorophyll molecules, as well as to absorb photon excess under high light in order to protect the photosynthetic apparatus from photo-damage [4].

Carotenoids, which are among the most potent antioxydants, have received much attention as potential anti-cancer and anti-inflammatory agents. Several carotenoids are produced industrially for their antioxidant properties and/or as staining agents in aquaculture. Astaxanthin from *Haematococcus pluvialis* and *Chlorella zofigiensis* was shown to significantly reduce the presence of free radicals and thus counteract oxidative stress [5,6]. Astaxanthin also has potential in the treatment of cardiovascular diseases [7]. β-carotene, which accumulates in *Dunaliella salina* at up to 14% dry weight [8], is a vitamin A precursor in humans. Molecules derived from β-carotene, like lutein and violaxanthin, have anti-inflammatory properties [9]. Lutein is also used in eye therapies; in particular to treat age-related macular degeneration [10]. Although carotenoid activities rely mainly on their antioxidant properties, the diversity of their chemical structures, with more than 700 reported molecules, opens a vast field of investigation and potential applications for these molecules.

The production of carotenoids at an industrial scale requires: (i) the optimization of culture conditions for carotenoid biosynthesis and accumulation and (ii) the selection of overproducing strains. Various stresses induce carotenoid accumulation. Cordero et al. (2012) demonstrated that the regulation of the carotenogenic pathway of *Chlorella zofingiensis* was influenced by light and nitrogen [11]. Both light and nitrogen-starvation stress enhance the accumulation of β-carotenoid, such as astaxanthin, canthaxanthin or zeaxanthin. This stress, however, had also an antagonistic effect on the production of α-carotenoids. Temperature stress of 40 °C was also shown to induce β-carotene accumulation up to 5% of dry weight in *Tetraselmis* sp. [12]. Finally, high salinity induces lipid and carotenoid production in *Chlorella zofingiensis* but reduces growth rates, leading to biomass decrease [13,14].

Metabolic engineering provides an alternative for selecting strains that overproduce carotenoids of interest. A zeaxanthin over-expressing strain was obtained by mutagenesis in *Dunaliella*. Phytoene synthase (*psy*) is a key enzyme involved in the first step of carotenoid synthesis. The over-expression of *Chlorella psy* enhances the accumulation of violaxanthin and lutein in *Chlamydomonas* and of β-carotene in *Scenedesmus* sp. [11,15]. The heterologous expression of the lycopene β-cyclase/lycopene ε-cyclase/light-harvesting complex-fusion protein from the green alga *Ostreococcus lucimarinus* allowed the modulation of the ratio between α- and β-pathways in bacteria. This enzyme may, therefore, be a target of interest for metabolic engineering of carotenoid synthesis in *Ostreococcus* cells [16,17].

The genus *Ostreococcus* (Mamiellophyceae) was originally discovered in the Thau lagoon and described as the smallest free-living eukaryotes [18]. Since the early sequencing of the *Ostreococcus tauri* OTTH595 strain, genetic transformation was implemented including gene targeting by homologous recombination [17,19,20]. *Ostreoccocus* has become a model organism to address biological questions such as circadian clock architecture [20,21], cell-cycle regulation [22] or the regulation of iron homeostasis [23]. Picophytoplanktonic eukaryotes belonging to Mamiellophyceae (*Ostreococcus, Micromonas* and *Bathycoccus*), have a worldwide distribution representing the 4th most abundant group in sequence numbers in the TARA Ocean Cruise [24]. Several strains of *Ostreococcus* have been sequenced [25] and comparative analysis has revealed the existence of “strains or ecotypes”, which are adapted to specific ecological niches with respect to light intensity (depth) or iron requirements [23,26,27].

*Ostreococcus* cells present several traits of interest as a source of high added-value molecules for biotechnologies. In addition to a fast growing rate, this microalga contains high amounts of long-chain polyunsaturated omega fatty acids such as DHA [28,29]. *Ostreococcus* cells are also a rich source of carotenoids including those that are widely distributed such as violaxanthin, antheraxantin or zeaxanthin, or Mamiellophyceae-specific carotenoids such as micromonal, uriolide or prasinoxanthin [27].

We have studied the potential of three *Ostreococcus* strains (OTTH595, RCC809 and RCC802) to produce specific carotenoids in response to light, temperature and salinity stress in batch conditions. Growth rates were also determined to evaluate the potential of these strains to produce carotenoids in continuous culture maintained in an exponential phase of growth.

## 2. Results

### 2.1. Growth Rates of Ostreococcus Strains under Abiotic Stresses

Three *Ostreococcus* strains were selected for comparative analysis of carotenoid production under light, temperature or salinity stress conditions (Table 1). Two strains (RCC802, RCC809) are oceanic; the third strain is the original strain of *Ostreococcus* (*Ostreococcus tauri*, OTTH595) that was isolated from the Thau Lagoon [18]. Both RCC802 (Mediterranean) and RCC809 (Atlantic tropical) come from deep environments where light is limited; RCC809 was described as a low-light strain [26,30]. Based on sequence homology RCC802 is an *Ostreococcus lucimarinus* strain, a species described as “high-light adapted” [23,27]. These three strains differ in term of temperature niches. The Thau lagoon (OTTH595) is a shallow environment displaying dramatic fluctuation in temperature over the year (4 °C to 30 °C) whereas RCC802 and RCC809 were isolated from the environment where the temperature is fairly stable over the year. Similarly, the salinity of the Thau lagoon is subject to large variations because of flood events and strong evaporation in summer. It should be noted also that the three strains belong to different phylogenetic clades (A, B and C).

We first determined the ability of *Ostreococcus* strains to survive and grow in response to changes in light, salinity and temperature conditions (Table 2). In all experiments cultures cells were exposed to 12 h/12 h light/dark cycle, the light period mimicking realistic solar curves of irradiation (see Material and Methods). The standard light condition corresponded to a light intensity of 150 μmol quanta m^−2^·s^−1^, at solar noon, temperature of 20 °C and salinity of 36 g·L^−1^. Cultures were acclimated for one week in each light/temperature conditions and refreshed before measuring the growth rate. Depending on the stress applied, differences were observed between the three strains. While both OTTH595 and RCC809 grew between 12 °C and 32 °C, RCC802 displayed lower temperature preferences between 10 °C and 27.5 °C. OTTH595, unlike RCC802 and RCC809, grew under low salinities (7.5 g·L^−1^ of NaCl), while RCC802 did not survive at salinities below 15 g·L^−1^ of NaCl. All three strains tolerated high salinities of 65 g·L^−1^ of NaCl. Under low light (50 μmol quanta m^−2^·s^−1^) growth was detected only in OTTH595. All strains except RCC802 were able to grow at light intensities up to 1500 μmol quanta m^−2^·s^−1^ at solar noon. The highest specific growth rates were observed under the standard light conditions for OTTH595 (1.66 d^−1^), RCC809 (1.64 d^−1^) and RCC802 (1.52 d^−1^).

### 2.2. Total Carotenoid Content under Various Stress Conditions

Cultures were grown in 50 mL batches, as described in the above section, under various conditions. Carotenoids were quantified by high-performance liquid chromatography (HPLC) (Table 3). In the standard culture condition, RCC809 had the highest content of total carotenoid (250.5 µg·L^−1^). In OTTH595, the highest concentrations were found under high temperature (279 µg·L^−1^), high light (266.2 µg·L^−1^), and low salinity (265.4 µg·L^−1^) and the lowest content was detected under low light (33.3 µg·L^−1^) and low temperature (18.5 µg·L^−1^) with a more than 15-fold reduction between the high-temperature and low-temperature conditions. In RCC802, the highest concentration of carotenoids was obtained at low salinity (431.1 µg·L^−1^). We also estimated the concentration of carotenoids per cell to take into account the differences in growth rates between stress conditions (Table 3). The highest cellular concentrations of total carotenoids were observed at 27.5 °C for OTTH595 (11.09 pg·cell^−1^) and at 30 °C for RCC809 (7.70 pg·cell^−1^), but for this latter strain this was not significantly higher than in the standard condition (6.23 pg·cell^−1^) in a Student’s *t*-test (*p*-value < 0.05). The highest cellular concentration of carotenoids occurred under low salinity in RCC802, (9.27 pg·cell^−1^) corresponding to a 2-fold increased compared to standard conditions.

The relative concentrations of α- and β-carotenoids are shown in Figure S1. The β-carotenoid relative content increased and conversely the α-carotenoid relative content decreased under high light irradiances (Figure S1A). The increase of β-carotenoid was more pronounced in OTTH595 and RCC809 (from 39% to 52% and from 40% to 51%, respectively) than in RCC802 (from 41% to 45%). High or low salinity had either a moderate effect on the relative content in α- and β-carotenoids in OTTH595 or no significant effect in RCC809, but low salinity favored the α-pathway in RCC802 (Figure S1B). An increase in temperature from 12 °C to 30 °C had similar effects on α- vs β-carotenoids content for OTTH595 and RCC802 with a decrease of the β-pathway (from 39% to 34% in OTTH595 between 12 °C and 30 °C and from 41% to 39% in RCC802 between 12 °C and 27.5 °C) relative to the α-pathway increase (Figure S1C).

### 2.3. Effect of Stress Conditions on Carotenoid Profiles

**Figure 1:**
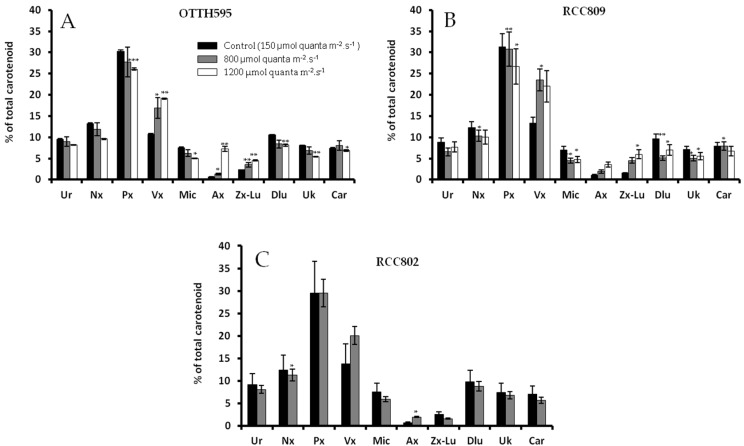
Effect of light intensity on the relative proportion of each carotenoid in *Ostreococcus* OTTH595 (**A**), RCC809 (**B**) and RCC802 (**C**). Cultures were exposed to 12:12 light dark cycles of intensities of 150 (control), 800 and 1200 µmol quanta m^−2^·s^−1^ at solar noon. Each carotenoid is expressed as percentage of the sum of uriolide (Ur), neoxanthin (Nx), prasinoxanthin (Px), violaxanthin (Vx), micromonal (Mic), antheraxanthin (Ax), zeaxanthin + lutein (Zx + Lu), dihydrolutein (Dlu), α + β carotene (Car) and one unknown carotenoid (Uk). Asterisks show significance in the Student’s *t*-test (* *p* < 0.05; ** *p* < 0.01, *** *p* < 0.001).

We investigated the effect of light intensity on the carotenoid profiles inferred from HPLC analysis (Figure 1). Ten carotenoids were identified, in all three strains. Prasinoxanthin was the most abundant molecule, representing between 30% and 35% of total carotenoid content. Increasing light intensity resulted in a dramatic accumulation of xanthophylls, including violaxanthin, antheraxanthin and zeaxanthin, in the three strains. This changes between standard light and high light were more pronounced for violaxanthin in RCC809 (from 13% to 23% of total carotenoid) and for antheraxanthin in OTTH595, (from 0.62% to 7.22% of total carotenoid). Zeaxanthin and lutein also increased in response to light in these two strains but not in RCC802. Finally the xanthophyll neoxanthin did not change in response to light in any of the strains.

Temperature and salinity had almost no impact on carotenoid profiles in batch cultures (Figures S2 and S3). Only, violaxanthin content decreased slightly with temperature level in all the strains. When looking at the cellular content, prasinoxanthin was the most abundant carotenoid in all strains, reaching under high temperature up to 2.36 pg·cell^−1^ and 2.51 pg·cell^−1^ in OTTH595 and RCC809, respectively, and 3.19 pg·cell^−1^ under low salinity in RCC802 (Table S3). Violaxanthin increased strongly in response to high light, high temperature and low salinity in OTTH595 (0.67, 0.66 and 0.70 pg·cell^−1^, respectively) but it was maximal only under low salinity in RCC802 (1.21 pg·cell^−1^) and it remained fairly constant under all stress conditions in RCC809. Under high light, antheraxanthin levels were 25 fold higher in OTTH595 (0.25 pg·cell^−1^) compared to standard conditions, but its level changed only by 2 fold in RCC802 and RCC809 (0.13 pg·cell^−1^ and 0.07 pg·cell^−1^, respectively).

### 2.4. Effect of Stress Conditions on Carotenoid Productivities

**Figure 2:**
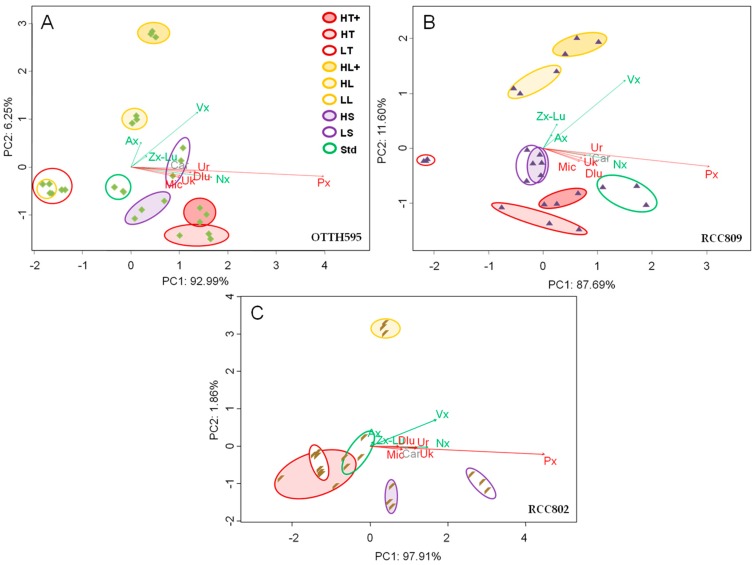
Redundancy analysis (RDA) of carotenoid productivities in batch cultures of OTTH595 (**A**), RCC809 (**B**) and RCC802 (**C**), respectively. Red, purple, yellow correspond to temperature-, salinity- and light-stress conditions. Green is the standard control condition. Color intensity increases with the intensity of the applied stress. The length of each arrow represents the relative influence of each carotenoid of the beta (red) or alpha (green) pathway on group separations.

The productivity of each carotenoid was determined in batch cultures after 7 days, i.e., either during or at the end of the exponential phase of growth depending on the stress applied. To compare carotenoid profiles in the three strains under various stress conditions, a multivariate analysis was performed (Figure 2). The first component accounted for most of the variability observed under the different stress conditions (92.99% for OTTH595, 87.69% for RCC809 and 97.91% for RCC802). Redundancy analysis (RDA) revealed that prasinoxanthin was the main carotenoid influencing group separation along the first component. Together the first and the second component accounted for 99% of the observed variability. Violaxanthin and to some extent antheraxanthin influenced the separation of samples by the second component, although this was less pronounced for RCC802. Analysis of variance using distance matrices (ADONIS) was performed to create a matrix of similarity to compare the different groups corresponding to biological triplicates of each strain under specific stress conditions. Most groups were statistically different in similarity percentage (SIMPER) analysis (*p*-value < 0.001). However differences were noted between the different strains depending on the stress condition applied. High temperature and low salinity were the main parameters influencing carotenoid productivity in OTTH595 (Figure 2A), and in RCC802 (Figure 2C), respectively, but the productivity was not impacted by stress in RCC809 (Figure 2B). In contrast, high light influenced violaxanthin productivity in all strains, and antheraxanthin in OTTH595.

The productivities of 148 carotenoids of 240 quantified (24 samples) were found to be significantly different from the control (*p*-value ≤ 0.05 in a paired parametric Shapiro–Wilk *t*-test). Under standard culture conditions, the prasinoxanthin productivity in the batch culture was 7.74 µg·L^−1^·d^−1^; 11.19 µg·L^−1^·d^−1^ and 7.09 µg·L^−1^·d^−1^ for OTTH595, RCC809 and RCC802, respectively. Under specific stress conditions, significant increases were observed, reaching up to 12.35 µg·L^−1^·d^−1^ at high temperature for OTTH595 and 21.15 µg·L^−1^·d^−1^ at low salinity for RCC802, These results confirmed that low salinity and high temperature stimulate prasinoxanthin production in RCC802 and OTTH595, respectively (Table S1). Similarly, violaxanthin productivity increased in response to high light conditions for the three strains. Noteworthy antheraxanthin productivity was strongly enhanced under high light conditions (2.72 µg·L^−1^·d^−1^) in OTTH595 compared to control condition (0.16 µg·L^−1^·d^−1^).

**Figure 3:**
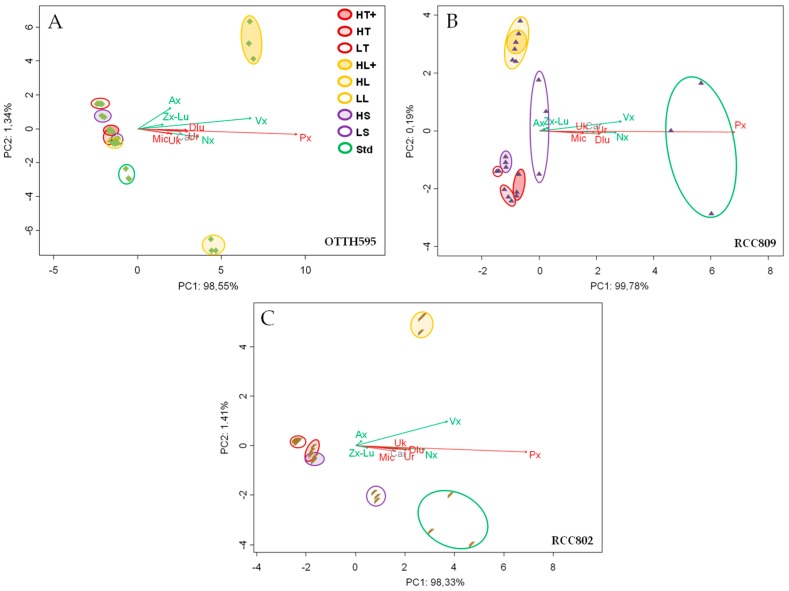
Redundancy analysis (RDA) of carotenoid theoretical maximal productivities of OTTH595 (**A**), RCC809 (**B**) and RCC802 (**C**), respectively. Red, purple, yellow correspond to temperature-, salinity- and light-stress conditions. Green is the standard control condition. Color intensity increases with the intensity of the applied stress. The length of each arrow represents the relative influence of each carotenoid of the beta (red) or alpha (green) pathway on group separations.

We next estimated the theoretical maximal productivity in the exponential phase of culture based on growth rate in each stress condition, as determined earlier (Table S2). Multivariate RDA analysis was performed to compare the different conditions (Figure 3). Under batch conditions, prasinoxanthin and violaxanthin/antheraxanthin were the main pigment correlated to the variability of the first and second component, respectively. Carotenoid productivities, in particular prasinoxanthin, were influenced by high light, in OTTH595 (Figure 3A), and they were not affected much by stress in RCC809 (Figure 3B). In RCC802, the productivity was influenced by high light and to some extent by low salinity (Figure 3C).

In agreement with these results, we noted in RCC802 a maximal productivity of prasinoxanthin under standard conditions (55.08 µg·L^−1^·d^−1^) and RCC809 (52.24 µg·L^−1^·d^−1^), respectively, and under high light in OTTH595 (100.77 µg·L^−1^·d^−1^) (Table S2). Violaxanthin productivity reached up to 73.81 µg·L^−1^·d^−1^ in OTTH595 when exposed to high light, while in RCC802 and RCC809 the maximal productivity was higher under standard conditions (23.55 and 28.09 µg·L^−1^·d^−1^, respectively). Antheraxanthin maximal productivity (27.91 µg·L^−1^·d^−1^.) was the highest in OTTH595 (compared to 0.57 and 2.82 µg·L^−1^·d^−1^ maximal productivities in RCC809 and RCC802, respectively).

RDA was also performed for the three strains, considering α- and β-carotenoids globally (Figure S4). The α-pathway influenced the variability of the first component while the β-pathway influenced the second component also. The low salinity had a moderate impact on the α-carotenoid productivity in RCC802 and the high light strongly influenced the α- and β-pathway in OTTH595, in particular when considering the maximal productivity (Figure S4).

**Figure 4:**
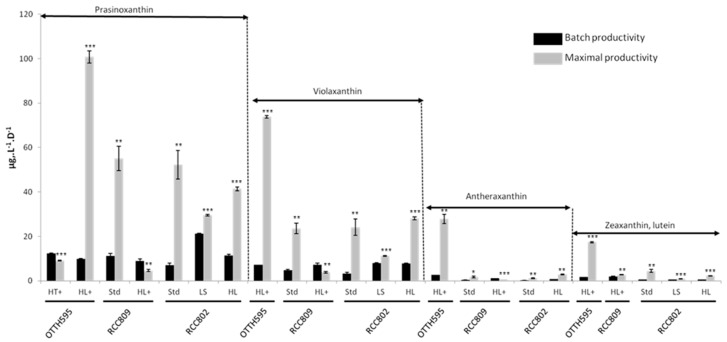
Comparizon of batch and theoretical maximal productivities of prasinoxanthin, violaxanthin, antheraxanthin and zeaxanthin + lutein in OTTH595, RCC809 and RCC802. Light stress of 800 µmol quanta m^−2^·s^−1^ (HL), 1200 µmol quanta m^−2^·s^−1^ (HL+). Salinity stress of 15g·L^−1^ NaCl (LS). Temperature stress of 30 °C (HT+). Standard control condition (Std). Each carotenoid is expressed as µg·L^−1^·d^−1^. Data are means ± standard deviation of three replicates, and asterisks show significance between the batch and the optimal productivity in a Student’s *t*-test (* *p* < 0.05; ** *p* < 0.01; *** *p* < 0.001).

Figure 4 summarizes the optimal conditions for the production of the main carotenoids either in batch or extrapolated under the exponential phase condition. The low salinity condition was optimal for the maximal production of prasinoxanthin in RCC802 in the batch. The high light condition, in contrast, was more favorable for prasinoxanthin maximal production in OTTH595. Violaxanthin productivity was highest under high light and low salinity in RCC802 in batch conditions. The theoretical maximal productivity for violaxanthin, antheraxanthin and zeaxanthin + lutein, however, was the highest in OTTH595 exposed to high light.

## 3. Discussion

In this study we compared the potential of three strains of the genus *Ostreococcus* to produce carotenoids under a wide range of light, salinity and stress conditions. Carotenoid profiles similar to those previously described were detected by HPLC [31]. Ten pigments were resolved including some that were specific such as prasinoxanthin, uriolide, micromonal and one unknown, as well as more common carotenoids such violaxanthin, antheraxantin, zeaxanthin + lutein, neoxanthin, dihydrolutein and α/β carotene. Under our standard batch conditions of light (150 μmol quanta m^−2^·s^−1^, temperature (20 °C) and salinity (36 g·L^−1^ of NaCl) the cellular concentrations of total carotenoid were variable between strains (4.62, 2.01 and 6.24 pg·cell^−1^ for RCC802, OTTH595 and RCC809, respectively). The carotenoid productivities, however, were similar in 7-day batch culture for, RCC802, OTTH595 and RCC809 (167.613 µg·L^−1^, 179.44 µg·L^−1^, 250.50 µg·L^−1^, respectively) because of differences in growth rates between the three strains (Table 2).

We observed that the carotenoid content is modulated quantitatively and qualitatively by abiotic stresses with strain-specific responses. The differential effect of high light stress was noted between the strains with respect to the quantitative and qualitative content of the carotenoids (Figure 2, Table 3 and Table S3). In OTTH595, a 7-fold induction was observed in total cellular carotenoid content between low light and high light, but in other strains the total cellular content of carotenoids decreased under high light (Table 3). Higher growth rates were also observed under high light in OTTH595. As a result, despite cellular carotenoid content being lower under high light (3.52 pg·cell^−1^) than under high temperature (11.09 pg·cell^−1^), production in 7-day batches were almost equivalent between the two conditions (266.256 µg·L^−1^ vs. 278.981 µg·L^−1^). OTTH595 is a lagoon strain that was described as a high-light “ecotype”, whereas RCC809 and RCC802 are deep strains and considered low-light ecotypes [26,27]. These differences in light niches may account for the differences observed in the light-dependent regulation of carotenoid synthesis. Carotenoids are located within the thylakoid membrane inside the chloroplast where they are associated with protein-like light-harvesting complexes (LHCs) which absorb light and transfer its energy to chlorophyll, triggering the photochemical events of photosynthesis. Xanthophylls play an important role in the protection of the photosynthetic apparatus against photo-oxidative damage. The conversion of violaxanthin into zeaxanthin, in the violaxanthin cycle, is a well-known mechanism involved in photoprotection [32]. Violaxanthin dissipates the excess of PSII excitation energy into heat through non-photochemical quenching. We were not able to quantify zeaxanthin, since lutein and zeaxanthin cooeluted in our HPLC analysis, as previously described [31]. Antheraxanthin is an intermediate in the violaxanthin cycle. In OTTH595, violaxanthin increased in response to high light by a two-fold factor (Table S3). Differences in the levels of antheraxanthin were observed between low and high light, with a 25-fold induction in OTTH595 but only a 2-fold induction in the two other strains. The conversion of antheraxanthin into zeaxanthin is, therefore, likely to be less efficient in OTTH595. Although the biological meaning of antheraxantin accumulation in OTTH595 under high light is not clear, our observation suggests that *Ostreococcus tauri* could be an interesting source of antheraxanthin as well as zeaxanthin + lutein (Figure 4).

High temperature was the major factor enhancing carotenoid content in OTTH595 with 11.09 pg·cell^−1^, while low salinity strongly stimulated carotenoid content in RCC802 (9.26 pg·cell^−1^). These results suggest that carotenoids could contribute to the adaptation of strains to specific environmental niches. RCC802, for example, does not grow below salinities of 15 g·L^−1^ NaCl and accumulates high amounts of carotenoids under this low-salinity condition. The induction of carotenogenesis by salinity has been documented in other microalgae such as *Haemotococcus* or *Dunaliella* [33,34]. Carotenoid accumulation, in contrast, is enhanced under high temperature in OTTH595, a strain that was isolated from the Thau Lagoon, an environment marked by large temperature fluctuations (Table 3 and Table S3). This effect of temperature has been reported in other microalgae strains such as *Chlorella vulgaris* [35]. Prasinoxanthin, the main carotenoid pigment in Prasinophyceae, accounted the most for the increased carotenoid productivity in response to low-salinity and high-temperature stress in RCC802 and OTTH595 (Figure 2). It should be noted, however, that under these conditions the cellular content of all carotenoids increased (Table S3). No specific induction of the β-pathway, in particular xanthophylls, was observed, unlike under high light. This suggests that the primary function of carotenoids under these salinity and temperature conditions is not photoprotection. The global induction of carotenoids in RCC802 and OTTH595 in response to low salinity and high temperature suggest that their accumulation is, rather, a response to oxidative stress arising from either temperature or osmotic stress. Such an induction of carotenogenesis, in particular lutein, is triggered in *Muriellopsis* sp. by reactive oxygen species induced by high temperature [36].

*Ostreococcus* strains contain specific Prasinophyceae-specific carotenoids such as prasinoxanthin, micromonal or uriolide and one unknown molecule representing more than 50% of total carotenoids, for which no biological activity have been described. As carotenoids are strong antioxidants, most of them reduce oxidative stress associated with diseases such as diabetes, cancer and eye pathologies [37]. Microalgal carotenoids including fucoxanthin, cantaraxanthin or astaxantin have anti-proliferating capacity. Astaxanthin, in particular, has been shown to inhibit the proliferation of bladder [38], colon [39] or leukemia cells [40]. It would be of great interest to test the activity of *Ostreococcus* prasinoxanthin, micromonal or uriolide. Several carotenoids of the beta family including zeaxanthin, lutein, or violaxanthin exhibit anti-inflammatory activities. Under high light stress, OTTH595 accumulates up to around 20% of violaxanthin and OTTH595 up to 7% of antheraxanthin, but antheraxanthin and zeaxanthin + lutein levels remained low in RCC809 (3.7% and 6%). Comparison of the activities of carotenoid extracts of the different strains grown under specific condition (e.g., OTTH595 and RCC809 under high light) could give insights into the biological activity of these carotenoid extracts.

In our experiments, carotenoids were quantified at the end of exponential phase. Under these conditions RCC802 appeared to be the most interesting strain for the production of total carotenoids. Extrapolation of productivities based on growth rates in the exponential phase, however, suggest that OTTH595 could potentially produce more carotenoids under high light because of higher growth rates. In the future, it would be interesting to combine stresses such as high light and salinity to improve carotenoid productivity. The effect of nitrogen starvation could also be tested alone or in combination with the other stresses [41]. Considering industrial production of carotenoids in industrial photobioreactors exposed to natural sunlight, our results suggest that OTTH595 would be the most interesting strain, in particular in summer when light intensity and temperature are high. In winter, however, when light intensity is reduced, RCC809 or RCC802 under low salinity conditions would be less expensive to produce.

## 4. Materials and Methods

### 4.1. Algal Culture and Harvesting

Microalgal strains of *Ostreococcus tauri* (OTTH595), *Ostreococcus lucimarinus* (RCC802) and *Ostreococcus* sp. (RCC809) were obtained from Roscoff culture collection. The strains were cultivated in 100 mL flasks or 96-deep well plates (Nunc, Perki Elmer, Hessen, Germany), in filtered artificial seawater (24.55 g·L^−1^ NaCl, 0.75 g·L^−1^ KCl, 4.07 g·L^−1^ MgC_l2_·6H_2_O, 1.47 g·L^−1^ CaCl_2_·2H_2_O, 6.04 g·L^−1^ MgSO_4_·7H_2_O, 0.21 g·L^−1^ NaHCO_3_, 0.138 g·L^−1^ NaH_2_PO_4_ and 0.75 g·L^−1^ NaNO_3_) supplemented with trace metals and vitamins. Cultures were maintained under constant gentle agitation at 20 °C in an orbital platform shaker (Heidoph shaker and mixer unimax 1010) under a 12-h light/12-h dark cycle. Realistic sunlight irradiation curves were applied during the light period with a maximum of 150 µmol quanta m^−2^·s^−1^ at solar noon in temperature-controlled incubators (Panasomic MIR-154-PE). For temperature, light and salinity experiments, algal cells inoculated in triplicate at 1 million cells·mL^−1^ in 50 mL flasks or deep well microplates (1.5 mL wells) were first acclimated for 7 days in the tested condition. Cells were refreshed at 1 million cells·mL^−1^ in deep wells for growth rate experiments or 50 mL flasks for carotenoid analysis. Experiments were first performed to explore growth rates under different salinities (15–60 g·L^−1^ of NaCl), temperature (12–30 °C) and light intensities (50–1200 µmol quanta m^−2^·s^−1^).

For growth experiments, all samples from biological triplicate (20 µL) were fixed with 0.25% glutaraldehyde (Sigma-Aldricht, St Louis, MO, USA) for 15 min at room temperature, before being frozen on dry ice. Samples were stored at −20 °C until cell counting. The cell number was determined using a BD accury C6 flow cytometer. The growth rate was determined as described below.

Based on growth rates the following conditions were selected for carotenoid analysis. For light experiments, 12 h light:12 h dark cycles were applied with light intensities at solar noon of 50 µmol quanta m^−2^·s^−1^ for low light (LL) , 150 µmol quanta m^−2^·s^−1^ for standard light, 800 µmol quanta m^−2^·s^−1^ (HL) and 1200 µmol quanta m^−2^·s^−1^ (HL+) for high light depending on the light tolerance of the strain. For temperature experiments, temperature of 12 °C (LT−), 15 °C (LT), 27.5 °C (HT) and 30 °C (HT+) were used depending on the strain tolerance. For salinity experiments, conditions of either low salinity (15 g·L^−1^ of NaCl) of high salinity of 50 g·L^−1^ NaCl (HS50) or 60 g·L^−1^ NaCl (HS60) depending on the strain tolerance. At the end of the culture, the 50 mL of culture was filtered onto a cellulose membrane GF/F 25 mm, (pore size 0.7 µm filters; Dominique Dutscher: 036334B) in darkness. Then filters were carefully dried to remove artificial sea water (ASW) before being transferred to cryotubes that were frozen in liquid nitrogen. Samples were stores at −80 °C prior to carotenoid analysis. Cells of each sample were also fixed for counting by flow cytometry.

### 4.2. Carotenoids Pigment Extraction and Analysis

The carotenoids on the GF/F filters were extracted at −20 °C in 3 mL methanol 100% and disrupted by sonication for 2 h. Separation and identification of the carotenoids were carried out using HPLC 1200 system (Agilent Technologies, Santa Clara, CA, USA). The methanol extract was mixed (1:1) with a buffer solution tetra-butylammonium acetate (TBAA) 28 mM and then, injected onto a narrow reversed-phase C8 Zorbax Eclipse XDB column (3 × 150 mm; 3.5 μm particle size) which was maintained at 60 °C. Elution of carotenoids was achieved within 28 min on a gradient between a solution (A) of TBAA 28 mM: methanol (30:70; v:v) and a solution (B) of 100% methanol according to the following program: (t (min); %B; %A), (0; 10; 90), (25; 95; 5), (28; 95; 5). Pigments were detected using a diode array detector, at 450 nm for carotenoids and 667 nm for chlorophyll a and its derivatives. Peak assignments and quantization were undertaken using carotenoid standards [42]. Chemicals and reagents of HPLC grade were purchased from Sigma Aldrich unless otherwise stated. Carotenoid content normalized to the cell number (determined by flow cytometry) was expressed in pg·g·L^−1^·cell^−1^. The productivity in 7-day batch culture (Pbatch) corresponding to the concentration of carotenoids produced by day was expressed in µg·L^−1^·d^−1^.

### 4.3. Determination of Growth Rates and Theoretical Maximal Productivities

For each culture condition the cell number was determined by flow cytometry daily, for a week. The growth rate in batch culture can be determined as Ln(*N*)/dt, where *N* is the cell concentration per mL (cells·mL^−1^) and *T* the time (days). The maximal growth rate (*µ_max_*) was determined on a graph expressing the neperian logarithm of cell concentration as a function of time of culture. *µ_max_* corresponded to the slope of the linear part of the growth curve (i.e., excluding the lag phase and the stationary phase).

$${µ}_{max}=\frac{Log\left(N_{fmax}\right)-Log\left(N_{0}\right)}{Log\left(2\right)\times \;T}$$

*N_fmax_* is number of cells at end of exponential growth phase; *N*_0_ is number of cells inoculated; *T* is the time (days) at the end of exponential growth phase.

In our batch conditions, cells were either at the end of the exponential phase of in the exponential phase, depending on the stress applied. To estimate the theoretical maximal cell number in continuous culture in which cells are maintained in the exponential phase of growth all the time (chemostat). We used the following equation:

$$N_{fmax}=e^{{µ}_{max}\times \;T_{batch}\;\times \;\ln\left(2\right)}$$

*T_batch_* is the duration of the batch culture (7 days in our experiments).

It follows that the theoretical maximal carotenoid productivity in continuous culture maintained in the exponential phase of growth (*P_max_*) is:

$$P_{max}=P_{batch}\;\times \;\left(\frac{N_{fmax}}{N_{f}}\right)$$

*N_f_* is the number of cells obtained at the end of the batch culture, *P_batch_* is carotenoid productivity in the batch determined experimentally.

### 4.4. Statistical Analysis

Analyses were performed in triplicate for each microalgae sample (*n* = 3). Means and standard deviations of the raw data and regression analysis of calibration samples were carried out using R statistic software. A test of normality, Shapiro–Wilkinson, (*p* > 0.05) followed by one way analysis of *t*-test variance was used to compare stressed conditions to the standard. Values were considered to be statistically significant when *p* < 0.05.

Multivariate redundant analyses (RDA) were conducted using the Vegan package to compare carotenoid productivities under different stress conditions. Analysis of similarity (ADONIS) was performed to compare groups. A resemblance matrix was built up based on pairwise Bray–Curtis distances. Similarity percentages (SIMPER), based on a non-parametric permutation procedure, were used to determine the contribution of each factor (i.e., each carotenoid) to the dissimilarities between groups.

## 5. Conclusions

We have identified 10 carotenoids in the three *Ostreococcus* strains. The results of our study highlight the potential of related strains originating from different environmental niches to obtain carotenoid content that is qualitatively and quantitatively different depending on the stress applied. They also illustrate that the growth rate in the exponential phase should be taken into account in order to extrapolate the strain’s ability to produce carotenoids in continuous culture for future large-scale production in a photobioreactor.

## Figures and Tables

**Table 1:** Description of *Ostreococcus* strains used in this study.

Strain Name	OTTH595	Eum16BBL_clonal	PROSOTOPE_44_clonal
#RCC	745	809	802
Species	*Ostreococcus tauri*	*Ostreococcus* sp.	*Ostreococcus lucimarinus*
Clade	C	B	A
Isolation date	03/05/1995	01/10/1991	18/09/1999
Latitude	+43°24′	+21°2′	+36°29′
Longitude	+3°36′	−31°8′	+13°19′
Depth (m)	Surface	105	65
Region	Thau lagoon, France	Tropical Atlantic Ocean	Sicily channel, Italy
Trophic level	Meso/eutrophic	Oligotrophic	Oligotrophic
Temperature	4 to 30 °C	18 to 20 °C	14 to 16 °C

**Table 2:** Growth rate obtained from *Ostreococcus* strains under temperature, light and salinity stress conditions. Growth rate is expressed as J^−1^. Data are presented as means ± standard deviation of three replicates. Asterisks show significance in Student’s *t*-test (* *p* < 0.05; ** *p* < 0.01, *** *p* < 0.001).

Condition		OTTH595	RCC809	RCC802
Temperature (°C)	10	0	0	0.18 ** ± 0.059
	12	0.31 ± 0.029	0.38 ** ± 0.008	0.37 ** ± 0.062
	15	0.41 ± 0.032	0.47 ** ± 0.051	0.50 ** ± 0.020
	20	0.82 ± 0.067	0.78 ± 0.017	0.84 ± 0.003
	25	0.74 * ± 0.030	0.59 ± 0.139	0.72 ± 0.139
	27.5	0.72 ± 0.085	0.55 ± 0.069	0.41 ± 0.206
	30	0.70 ± 0.041	0.46 *** ± 0.051	0
	32	0.59 ± 0.011	0.32 ** ± 0.017	0
Light (μmol quanta m^−2^·s^−1^)	50	0.49 ± 0.05	0	0
	150	1.66 ± 0.089	1.64 ± 0.086	1.52 ± 0.092
	500	1.65 ± 0.077	1.29 * ± 0.066	1.19 ± 0.082
	800	1.36 * ± 0.041	0.77 * ± 0.156	1.16 ± 0.350
	1200	1.37 * ± 0.026	0.72 ** ± 0.091	0
	1500	1.12 * ± 0.037	0.66 *** ± 0.043	0
	2000	0	0	0
Salinity (0–70‰)	0	0	0	0
	5	0	0	0
	7.5	0.64 * ± 0.123	0	0
	10	0.79 ± 0.039	0.83 ± 0.105	0
	15	0.83 * ± 0.057	0.91 ± 0.072	0.86 * ± 0.036
	36	0.96 ± 0.065	0.84 ± 0.005	1.12 ± 0.111
	57.5	0.68 * ± 0.018	0.67 * ± 0.048	1.07 ± 0.024
	60	0.65 * ± 0.018	0.51** ± 0.033	0.77 ± 0.222
	62.5	0.62 * ± 0.042	0.61* ± 0.074	0.52 ± 0.153
	65	0.44 ** ± 0.022	0.40 * ± 0.136	0.43 * ± 0.048
	70	0	0	0

**Table 3:** Total carotenoid content in 7-day batch cultures of *Ostreococcus strains* expressed either as µg·L^−1^ or by pg·cell^−1^ (means ± standard deviation). Stress conditions are detailed in main text. Asterisks show significance in Student’s *t*-test (* *p* < 0.05; ** *p* < 0.01; *** *p* < 0.001).

**Total Carotenoid (µg·L^−1^)**	**Treatments**										
**Microalgae**	**Control**	**LL**	**HL**	**HL+**	**LT−**	**LT**	**HT**	**HT+**	**LS**	**HS 50 g/L**	**HS 60 g/L**
**OTTH595**	179.443 ± 13.198	33.331 ** ± 2.492	168.001 ± 11.862	266.256 * ± 6.563	18.500 ** ± 2.899	60.000 ** ± 2.808	225.603 ± 8.454	278.981 ** ± 6.082	265.402 * ± 14.606	/	230.762 ± 37.176
**RCC 809**	250.50 ± 27.286	/	147.481* ± 32.157	233.55 ± 25.957	/	17.247 ** ± 1.522	128.403 * ± 31.104	124.815 * ± 9.689	142.657 * ± 13.689	/	141.464 * ± 7.367
**RCC 802**	167.613 ± 22.826	/	273.278 * ± 10.180	/	71.633 * ± 5.402	102.833 * ± 1.833	103.541 * ± 8.401	/	431.08 ** ± 7.605	277.513 ** ± 6.238	/
**Carotenoid Content (pg·cell^−1^)**	**Treatments**										
**Microalgae**	**Control**	**LL**	**HL**	**HL+**	**LT−**	**LT**	**HT**	**HT+**	**LS**	**HS 50 g/L**	**HS 60 g/L**
**OTTH595**	2.01 ± 0.029	0.515 *** ± 0.027	2.769 ± 0.36	3.520 *** ± 0.069	0.812 ** ± 0.127	0.800 ** ± 0.160	11.09 ** ± 0.776	7.559 ** ± 0.458	5.443 *** ± 0.206	/	3.063 * ± 0.229
**RCC 809**	6.236 ± 0.670	/	2.599 ** ± 0.327	3.470 * ± 0.563	/	0.320 ** ± 0.058	5.551 ± 0.313	7.703 ± 1.305	3.242 * ± 0.233	/	3.946 * ± 0.523
**RCC 802**	4.621 ± 1.223	/	3.567 ± 0.380	/	3.378 ± 0.148	1.376 ± 0.168	5.161 ± 0.420	/	9.268 * ± 1.203	3.514 ± 0.125	/

## Supplementary Materials

A supplementary dataset containing 4 figures and 3 tables is available online at http://www.mdpi.com/1660-3397/16/3/76/s1.
