# Supplementary material for: Comparative Analysis of Culture Conditions for the Optimization of Carotenoid Production in Several Strains of the Picoeukaryote Ostreococcus
Source: Mar Drugs. 2018 Feb 28;16(3):76. doi: 10.3390/md16030076 (PMC5867620; doi:10.3390/md16030076)
Supplement: Supplementary file 1 [file marinedrugs-16-00076-s001.pdf]

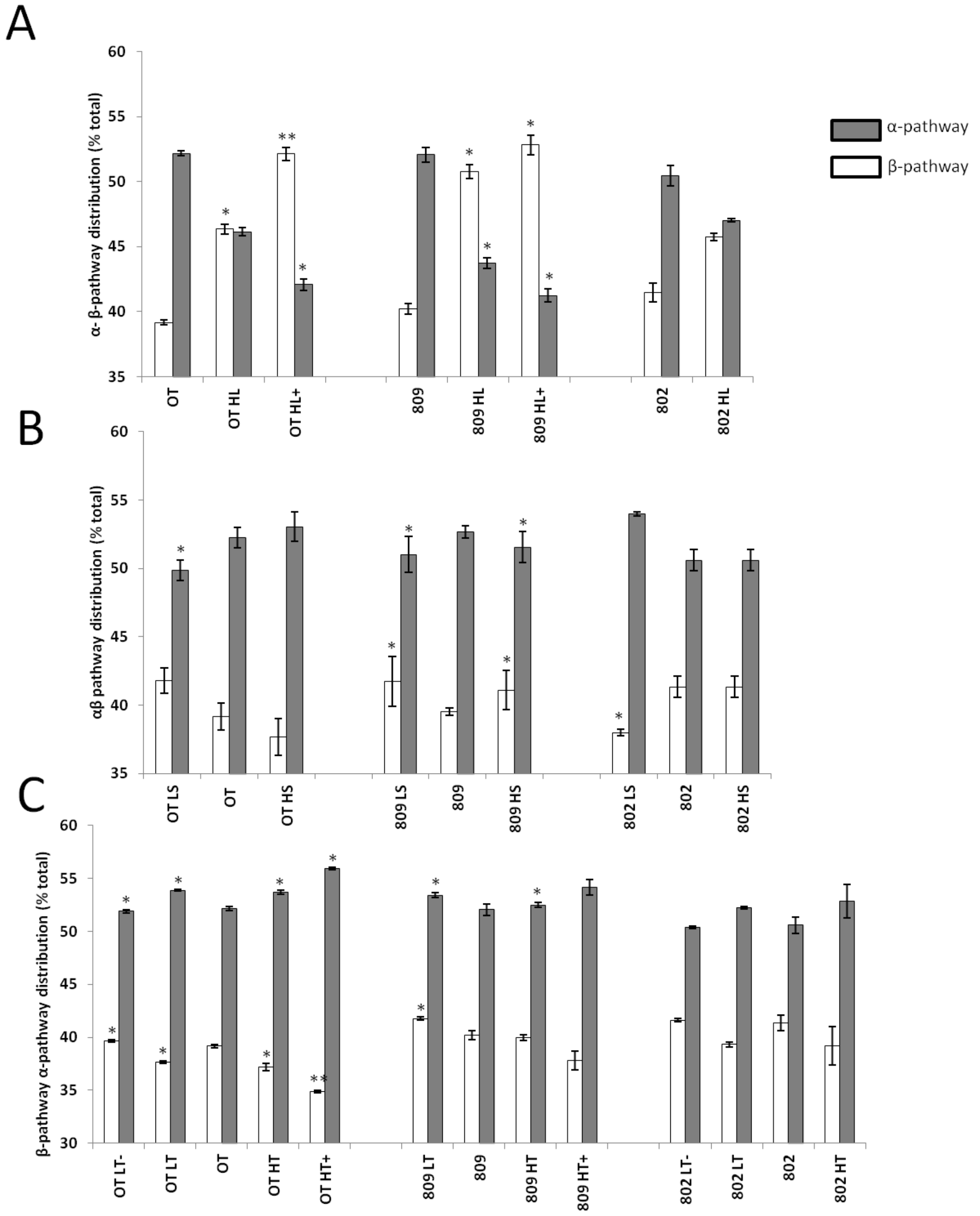

Figure S1 : Relative proportion of carotenoids of the α-pathway (grey box) and β-pathway (white box) in *Ostreococcus* OTTH595, RCC809 and RCC802 under **(A)** high light HL (800 µmol quanta $m^{-2}.s^{-1}$), HL+ (1200 µmol quanta $m^{-2}.s^{-1}$), **(B)** low salinity LS (15g.L^{-1} NaCl), high salinity HS50 (50 g.L^{-1} NaCl) HS60 (60g.L^{-1} NaCl), **(C)** low temperature LT- (12°C) and LT (15°C), high temperature HT (27°C) and HT+ (30°C). Data are presented as means ± standard deviation of three replicates, and asterisks show statistical significance in a Student *T*-test (*: $P < 0.05$; **$P < 0.01$;).

Table S1: Carotenoid productivity in 7 days batch culture of *Ostreococcus* OTTH595, RCC809 and RCC802. Each carotenoid is expressed as µg.L^{-1}.d^{-1} of uriolide, neoxanthin, prasinoxanthin, violaxanthin, micromonal, antheraxanthin, zeaxanthin+lutein, dihydrolutein, and α+β carotene. HL : 800 µmol quanta m^{-2}.s^{-1}; HL+ : 1200 µmol quanta m^{-2}.s^{-1} LT - : 12°C LT: 15°C; HT : 27.5°C; HT+ : 30°C; LS : 15g.L^{-1} NaCl, HS : 50 or 60 g.L^{-1} NaCl . Data are presented as means ± standard deviation of three replicates, and asterisks show statistical significance in a Student *T*-test (*: P < 0.05; **P < 0.01; ***; P< 0.001).

Strain	Condition	Uriolide (µg.L^{-1}.d^{-1})	Neoxanthin (µg.L^{-1}.d^{-1})	Prasinoxanthin (µg.L^{-1}.d^{-1})	Violaxanthin (µg.L^{-1}.d^{-1})	Micromonal (µg.L^{-1}.d^{-1})	Antheraxanthin (µg.L^{-1}.d^{-1})	Zeaxanthin+Lutein (µg.L^{-1}.d^{-1})	Dihydrolutein (µg.L^{-1}.d^{-1})	Unknown (µg.L^{-1}.d^{-1})	Carotene (µg.L^{-1}.d^{-1})
	Control	2.4±0.20	3.4±0.25	7.7±0.58	2.7±0.12	1.9±0.16	0.2±0.01	0.6±0.05	2.7±0.21	2.1±0.15	1.9±0.14
	HL	2.7±0.06	3.6±0.08	8.4±0.19	5.1**±0.06	1.9±0.05	0.4**±0.01	1**±0.02	2.5±0.10	2.1±0.06	2.4*±0.05
	HL+	3.1*±0.07	3.6±0.09	9.8*±0.27	7.2***±0.05	1.9±0.05	2.7**±0.20	1.7***±0.03	3.1*±0.08	2±0.04	2.6**±0.05
	LT-	0.2**±0.03	0.3***±0.05	0.8***±0.12	0.3***±0.05	0.2***±0.03	ND	5.10^{-2}**±0.01	0.3**±0.05	0.2***±0.03	0.2***±0.04
OTTH595	LT	0.8**±0.04	1.1**±0.05	2.7**±0.12	0.8***±0.03	0.7**±0.03	4.10^{-2}**±0.01	0.1**±0.01	0.9**±0.05	0.7**±0.03	0.6**±0.03
	HT	4.1**±0.43	5.4**±0.58	12.7**±1.28	3.1±0.46	3.2**±0.33	0.3**±0.03	0.8**±0.07	4.1**±0.41	3.4**±0.31	3.3**±0.35
	HT+	4**±0.06	5.1**±0.09	12.3**±0.17	3.4**±0.08	3.1**±0.06	0.5±0.15	1.1*±0.12	3.8**±0.08	3**±0.04	3.2**±0.01
	LS	4**±0.22	4.7**±0.20	11**±0.49	4.9*±0.59	2.9**±011	0.2*±0.03	0.7*±0.08	3.5**±0.10	2.9**±0.09	2.7**±0.13
	HS	3.3±0.54	4.4±0.64	9.5±1.34	3±0.84	2.8±0.44	0.1±0.01	0.5±0.12	3.6±0.58	2.8*±0.39	2.5**±0.35
	Control	3.1±0.27	4.4±0.34	11.2±0.79	4.8±0.33	2.5±0.21	0.4±0.05	0.5±0.03	3.4±0.30	2.5±0.18	2.8±0.21
	HL	0.9*±0.32	1.5*±0.48	4.5*±1.43	3.4± 0.95	0.6*±0.22	0.3±0.10	0.7±0.21	0.7*±0.22	0.7*±0.24	1.1*±0.34
	HL+	2.5±0.33	3.3±0.42	8.8±1.01	7.3±0.66	1.6±0.21	1.2*±0.06	2**±0.12	2.3±0.37	1.8±0.28	2.2±0.23
	LT-	ND	ND	ND	ND	ND	ND	ND	ND	ND	ND
RCC 809	LT	0.2**±0..01	0.3**±0.01	0.8**±0.04	0.4**±0.03	0.2**±0.01	2.10^{-2}±0.00	5.10^{-2}**±0.01	0.2**±0.01	0.1**±0.09	0.2** ±0.01
	HT	1.6**±0.40	2.3*±0.57	6*±1.40	1.6**±0.44	1.4**±0.33	0.2±0.07	0.3±0.17	1.8**±0.43	1.4*±0.30	1.6*±0.33
	HT+	1.6*±0.10	2.1*±0.14	5.9*±0.40	2.1*±0.36	1.2*±0.06	0.1±0.01	0.2*±0.01	1.6*±0.08	1.3*±0.07	1.7±0.15
	LS	1.7*±0.14	2.3*±0.21	6.4*±0.47	2.9±0.41	1.3*±0.13	0.3±0.04	0.5±0.07	1.7*±0.17	1.3*±0.15	1.7*±0.14
	HS	1.7*±0.07	2.3*±0.08	6.3*±0.28	3*±0.35	1.3*±0.04	0.3±0.04	0.4±0.05	1.7*±0.05	1.3*±0.03	1.6*±0.05
	Control	2.2±0.30	3±0.41	7.1±0.88	3.3±0.50	1.8±0.24	0.1±0.02	0.6±0.08	2.4±0.33	1.8±0.24	1.7±0.24
	HL	3.1±0.04	4.4±0.03	11.4*±0.22	7.8**±0.20	2.3±.006	0.8***±0.05	0.6±0.02	3.4±0.01	2.6±0.04	2.2±0.03
	HL+	ND	ND	ND	ND	ND	ND	ND	ND	ND	ND
	LT-	0.8*±0.06	1.2*±0.10	3*±0.22	1.6*±0.13	0.8*±0.06	0.1*±0.01	0.1**±0.01	1*±0.07	0.8*±0.06	0.6*±0.05
RCC802	LT	1.3*±0.03	1.8*±0.02	4.6*±0.04	1.9*±0.06	1.2*±0.02	0.2±0.01	0.2*±0.01	1.5*±0.02	1.2*±0.02	0.9*±0.02
	HT	1.3±0.09	1.8±.010	4.8±0.34	2±0.34	1.1±0.05	0.1±0.04	0.2±0.05	1.5±0.06	1.1±0.06	0.8±0.05
	HT+	ND	ND	ND	ND	ND	ND	ND	ND	ND	ND
	LS	5.6**±0.09	7.8**±0.14	21.1**±0.30	8**±0.09	4.1*±0.08	0.4**±0.01	0.7±0.03	5.9*±0.13	4.6**±0.11	3.5*±0.09
	HS	3.6*±0.07	5*±0.08	13**±0.20	4.7±0.27	3.1*±0.05	0.3*±0.03	0.2*±0.01	3.8*±0.06	3*±0.06	2.5±0.03

Table S2: Theoretical maximal carotenoid productivity in *Ostreococcus* OTTH595, RCC809 and RCC802. Each carotenoid is expressed as $\mu g.L^{-1}.d^{-1}$ of uriolide, neoxanthin, prasinoxanthin, violaxanthin, micromonal, antheraxanthin, zeaxanthin+lutein, dihydrolutein, and α+β carotene. HL : 800 µmol quanta $m^{-2}.s^{-1}$; HL+: 1200 µmol quanta $m^{-2}.s^{-1}$ LT -: 12°C LT: 15°C, HT : 27.5°C, HT+ : 30°C, LS : 15g.L^{-1} NaCl, HS : 50 or 60 g.L^{-1} NaCl . Data are presented as means ± standard deviation of three replicates, and asterisks show statistical significance in a Student *T*-test (*: P < 0.05; **P < 0.01; ***; P< 0.001).

Strain	Condition	Uriolide (µg.L^{-1}.d^{-1})	Neoxanthin (µg.L^{-1}.d^{-1})	Prasinoxanthin (µg.L^{-1}.d^{-1})	Violaxanthin (µg.L^{-1}.d^{-1})	Micromonal (µg.L^{-1}.D^{-1})	Antheraxanthin (µg.L^{-1}.d^{-1})	Zeaxanthin+Lutein (µg.L^{-1}.d^{-1})	Dihydrolutein (µg.L^{-1}.d^{-1})	Unknown (µg.L^{-1}.d^{-1})	Carotene (µg.L^{-1}.d^{-1})
OTTH595	Control	7.2±0.58	10±0.76	22.9±1.72	8.1±0.36	5.7±0.46	0.5±0.05	1.7±0.15	8±0.63	6.1±0.14	5.6±0.15
	HL	25.6**±0.62	34.1**±0.83	79.5**±1.80	48.4***±0.62	17.8**±0.49	3.8±*** 0.08	10***±0.17	24.1**±0.92	19.7**±0.55	23***±0.44
	HL+	31.5***± 0.64	37**±0.90	100.8**±2.74	73.8***±0.54	19.2**±0.50	27.9**±2.05	17.4***±0.35	31.4***±0.78	20.8**±0.45	26.6***±0.48
	LT-	5.10^{-2}**±0.01	0.1**±0.01	0.2**±0.02	7.10^{-2}***±0.01	4.10^{-2}**±0.01	ND	1.10^{-2}**±1.10^{-3}	0.1**±9.10^{-3}	4.10^{-2}**±6.10^{-3}	4.10^{-2}**±7.10^{-3}
	LT	8.10^{-2}**±7.10^{-3}	0.1**±0.01	0.3**±0.01	8.10^{-2}***±3.10^{-3}	7.10^{-2}**±3.10^{-3}	0.01**±2.10^{-4}	1.10^{-2}**±1.10^{-3}	0.1**±5.10^{-3}	0.1**±3.10^{-3}	0.1**±3.10^{-3}
	HT	3.2**±0.33	4.2**±0.43	9.7**±0.98	2.4**±0.35	2.5**±0.25	0.2**±0.02	0.6**±0.05	3.1**±0.31	2.6**±0.24	2.5**±0.27
	HT+	2.9**±0.04	3.8**±0.07	9.1**±0.13	2.5*±0.06	2.3**±.006	0.4±0.11	0.8*±0.09	2.8**±0.06	2.2**±0.03	2.3**±0.01
	LS	4.7*±0.26	5.5*±0.23	12.8*±0.57	5.6*±0.69	3.3*±0.13	0.3**±0.04	0.8**±0.10	4**±0.12	3.4**±0.11	3.1**±0.15
	HS	1**±0.20	1.4**±0.23	3**±0.49	0.9**±0.31	0.9**±0.16	3.10^{-2}**±4.10^{-3}	0.2**±0.05	1.1**±0.21	0.9**±0.14	0.8**±0.13
RCC 809	Control	15.5±1.89	21.6±2.42	55.1±5.57	23.5±2.35	12.4±1.51	1.8±0.37	2.7±0.20	16.9±2.09	12.5±1.29	14±1.48
	HL	1**±0.25	1.6**±0.37	4.9**±1.09	3.7*±0.73	0.7**±0.17	0.3*±0.08	0.7***±0.16	0.8**±.017	0.8**±0.19	1.3**±0.26
	HL+	1.3**±0.18	1.7**±0.22	4.7**±0.53	3.8**±0.35	0.8**±0.11	0.6*±0.03	1**±0.06	1.2**±0.19	1**±0.15	1.3**±0.12
	LT-	ND	ND	ND	ND	ND	ND	ND	ND	ND	ND
	LT	4.10^{-2}**±2.10^{-3}	5.10^{-2}**±0.01	0.1**±0.01	6.10^{-2}**±0.01	3.10^{-2}**±1.10^{-3}	1.10^{-2}*±5.10^{-4}	1.10^{-2}**±2.10^{-3}	4.10^{-2}**±1.10^{-3}	2.10^{-2}**±0.02	4.10^{-2}**±2.10^{-3}
	HT	0.8**±0.31	1.1**±0.45	2.8**±1.13	0.8**±0.35	0.6**±0.26	8.10^{-2}*±0.03	0.1**±0.10	0.9**±0.34	0.7**±0.27	0.8**±0.30
	HT+	1.4**±0.08	2**±0.13	5.4**±0.37	1.9**±0.33	1.1*±0.06	0.1*±0.01	0.2**±0.01	1.5**±0.07	1.2**±0.06	1.6**±0.14
	LS	3.2*±0.27	4.4**±0.40	12.1**±0.89	5.5**±0.77	2.5*±0.25	0.6*±0.08	0.9*±0.14	3.2*±0.33	2.5**±0.28	3.1**±0.27
	HS	0.6**±0.02	0.8**±0.03	2.1**±0.09	1**±0.11	0.4**±0.01	0.1*±0.01	0.1**±0.02	0.6**±0.02	0.4**±0.01	0.5**±0.02
RCC802	Control	16.2±2.23	21.9±3.00	52.2±6.47	24.1±3.69	13.3±1.80	1.1±0.17	4.5±0.60	17.4±2.44	13.2±1.83	12.4±1.80
	HL	11.4±0.16	15.9±0.11	41.4±0.80	28.1±0.73	8.4*±0.21	2.8*±0.19	2.3*±0.06	12.4±0.04	9.5±0.13	8*±0.09
	HL+	ND	ND	ND	ND	ND	ND	ND	ND	ND	ND
	LT-	0.2**±0.01	0.2**±0.02	0.6**±0.04	0.3**±0.03	0.2**±0.01	2.10^{-2}**±2.10^{-3}	2.10^{-2}**±2.10^{-3}	0.2**±1.10^{-3}	0.1**±0.01	0.1**±0.01
	LT	0.2**±4.10^{-3}	0.3**±0.00	0.7**±0.01	0.3**±0.01	0.2**±4.10^{-3}	3.10^{-2}**±1.10^{-3}	3.10^{-2}**±2.10^{-3}	0.2**±1.10^{-3}	0.2**±4.10^{-3}	0.1**±3.10^{-3}
	HT	1.7**±0.11	2.4**±0.13	6.2**±0.44	2.5**±0.44	1.4**±0.06	0.2**±0.05	0.3**±0.07	1.9**±0.08	1.4**±0.08	1**±0.07
	HT+	ND	ND	ND	ND	ND	ND	ND	ND	ND	ND
	LS	7.9*±0.12	10*±0.20	29.6*±0.42	11.2*±0.12	5.7*±0.11	0.5*±0.01	0.9*±0.05	8.2*±0.18	6.5*±0.15	4.9*±0.12
	HS	2**±0.04	2.7**±0.05	7.1**±0.11	2.5**±0.15	1.7**±0.03	0.2*±0.02	0.1**±0.01	2.1**±0.03	1.7**±0.03	1.4**±.001

Table S3: Carotenoid content in *Ostreococcus* species. Each carotenoid is expressed as pg.cell^{-1} in *Ostreococcus* OTTH595, RCC809 and RCC802. Each carotenoid is expressed as µg.L^{-1}.d^{-1} of uriolide, neoxanthin, prasinoxanthin, violaxanthin, micromonal, antheraxanthin, zeaxanthin+lutein, dihydrolutein, and α+β carotene. HL : 800 µmol quanta m^{-2}.s^{-1}, HL+: 1200 µmol quanta m^{-2}.s^{-1} LT -: 12°C LT: 15°C, HT: 27.5°C, HT+: 30°C, LS: 15g.L^{-1} NaCl, HS 50 or 60 g.L^{-1} NaCl . Data are presented as means ± standard deviation of three replicates, and asterisks show statistical significance in a Student *T*-test (*: P < 0.05; **P < 0.01; ***; P< 0.001).

Strain	Condition	Uriolide (pg.cell^{-1})	Neoxanthin (pg.cell^{-1})	Prasinoxanthin (pg.cell^{-1})	Violaxanthin (pg.cell^{-1})	Micromonal (pg.cell^{-1})	Antheraxanthin (pg.cell^{-1})	Zeaxanthin+Lutein (pg.cell^{-1})	Dihydrolutein (pg.cell^{-1})	Unknown (pg.cell^{-1})	Carotene (pg.cell^{-1})
OTTH595	Control	0.2±0.02	0.3±0.02	0.6±0.05	0.2±0.01	0.1±0.01	1.10^{-2}±1.10^{-3}	5.10^{-2}±4.10^{-3}	0.2±0.02	0.2±0.01	0.1±0.01
	HL	0.2±0.01	0.3±0.01	0.8±0.02	0.5**±0.01	0.2±5.10^{-3}	4.10^{-2}**±1.10^{-3}	0.1**±2.10^{-3}	0.2±0.01	0.2±0.01	0.2*±4.10^{-3}
	HL+	0.3*±0.01	0.3±0.01	0.9*±0.02	0.7***±5.10^{-3}	0.2±5.10^{-3}	0.2**±0.02	0.2***±3.10^{-3}	0.3*±0.01	0.2±4.10^{-3}	0.2*±4.10^{-3}
	LT-	8.10^{-2}**±0.01	0.1***±0.02	0.2***±0.04	0.1***±0.02	0.1***±0.01	ND	1.10^{-2}**±2.10^{-3}	0.1**±0.01	0.1***±0.01	0.1***±0.01
	LT	8.10^{-2}**±4.10^{-3}	0.1**±0.01	0.2**±0.01	0.1***±3.10^{-3}	0.1**±3.10^{-3}	1.10^{-2}±2.10^{-4}	1.10^{-2}**±1.10^{-3}	0.1**±4.10^{-3}	0.1**±3.10^{-3}	0.1**±3.10^{-3}
	HT	0.7**±0.07	0.9**±0.09	2.1**±0.21	0.5±0.08	0.5**±0.05	5.10^{-2}**±4.10^{-3}	0.1**±0.01	0.7**±0.07	0.5**±0.05	0.5**±0.06
	HT+	0.7**±0.01	1**±0.02	2.4**±0.03	0.7**±0.02	0.6**±0.01	0.1**±0.03	0.2**±0.02	0.7**±0.01	0.6**±0.01	0.6**±1.10^{-3}
	LS	0.6**±0.03	0.7**±0.03	1.6*±0.07	0.7*±0.09	0.4**±0.02	3.10^{-2}*±4.10^{-3}	0.1*±0.01	0.5**±0.02	0.4**±0.01	0.4**±0.02
	HS	0.3±0.05	0.4±0.06	0.9±0.13	0.3±0.08	0.3±0.04	1.10^{-2}±1.10^{-3}	5.10^{-2}±0.01	0.3±0.05	0.3*±0.04	0.2±0.03
RCC 809	Control	0.5±0.07	0.8±0.09	1.9±0.20	0.8±0.08	0.4±0.05	0.1±0.01	0.1±0.01	0.6±0.07	0.4±0.05	0.5±0.05
	HL	0.2*±0.04	0.3*±0.06	0.8*±0.18	0.6±0.12	0.1*±0.03	5.10^{-2}±0.01	0.1±0.03	0.1*±0.03	0.1*±0.03	0.2*±0.04
	HL+	0.3±0.04	0.3±0.04	0.9±0.11	0.8±0.07	0.2±0.02	0.1*±0.01	0.2*±0.01	0.2±0.04	0.2±0.03	0.2±0.02
	LT-	ND	ND	ND	ND	ND	ND	ND	ND	ND	ND
	LT	3.10^{-2}**±1.10^{-3}	4.10^{-4}*±0.01	0.1**±0.01	5.10^{-2}**±6.10^{-3}	2.10^{-2}**±1.10^{-3}	1.10^{-2}±3.10^{-4}	1.10^{-2}**±1.10^{-3}	3.10^{-2}**±1.10^{-3}	1.10^{-2}**±0.01	3.10^{-2}**±3.10^{-3}
	HT	0.5**±0.20	0.7**±0.29	1.8*±0.74	0.5**±0.23	0.4±0.17	5.10^{-2}±0.02	0.1±0.06	0.6±0.22	0.4±0.17	0.5±0.19
	HT+	0.7*±0.04	0.9*±0.06	2.5*±0.17	0.9±0.15	0.5±0.03	5.10^{-2}±0.01	0.1±0.01	0.7±0.03	0.5*±0.03	0.7±0.07
	LS	0.3*±0.02	0.4*±0.03	1*±0.08	0.5±0.07	0.2*±0.02	5.10^{-2}±0.01	0.1±0.01	0.3*±0.03	0.2*±0.02	0.3*±0.02
	HS	0.3*± 0.01	0.5*±0.02	1.2*±0.06	0.6*±0.07	0.3*±0.02	0.1±0.01	0.1±0.01	0.3*±0.01	0.3*±0.01	0.3*±0.01
RCC802	Control	0.4±0.06	0.5±0.08	1.3±0.16	0.6±0.09	0.3±0.05	3.10^{-2}±1.10^{-3}	0.1±0.02	0.4±0.06	0.3±0.05	0.3±0.05
	HL	0.3±4.10^{-3}	0.4±3.10^{-3}	1*±0.02	0.7±0.02	0.2±0.01	0.1*±5.10^{-3}	0.1±2.10^{-3}	0.3±1.10^{-3}	0.2±0.00	0.2±2.10^{-3}
	HL+	ND	ND	ND	ND	ND	ND	ND	ND	ND	ND
	LT-	0.2*±0.01	0.3*±0.02	0.7*±0.05	0.4*±0.03	0.2*±0.01	3.10^{-2}±2.10^{-3}	3.10^{-2}**±2.10^{-3}	0.2*±0.02	0.2*±0.01	0.1*±0.01
	LT	0.1*±2.10^{-2}	0.2*±0.00	0.4*±0.00	0.2*±0.01	0.1*±2.10^{-3}	1.10^{-2}±1.10^{-3}	2.10^{-2}*±1.10^{-3}	0.1*±0.00	0.1*±2.10^{-3}	0.1*±2.10^{-3}
	HT	0.5±0.03	0.6±0.04	1.7±0.12	0.7±0.12	0.4±0.02	5.10^{-2}±0.01	0.1±0.01	0.5±0.02	0.4±0.02	0.3±0.02
	HT+	ND	ND	ND	ND	ND	ND	ND	ND	ND	ND
	LS	0.8**±0.01	1.1**±0.02	3.2**±0.05	1.2**±0.01	0.6*±0.01	0.1**±1.10^{-3}	0.1±0.01	0.9*±0.02	0.7**±0.02	0.5*±0.01
	HS	0.3*±0.01	0.5*±0.01	1.2±0.02	0.4±0.03	0.3±5.10^{-3}	3.10^{-2}±3.10^{-3}	2.10^{-2}**±5.10^{-3}	0.3*±0.01	0.3*±0.01	0.2±2.10^{-3}

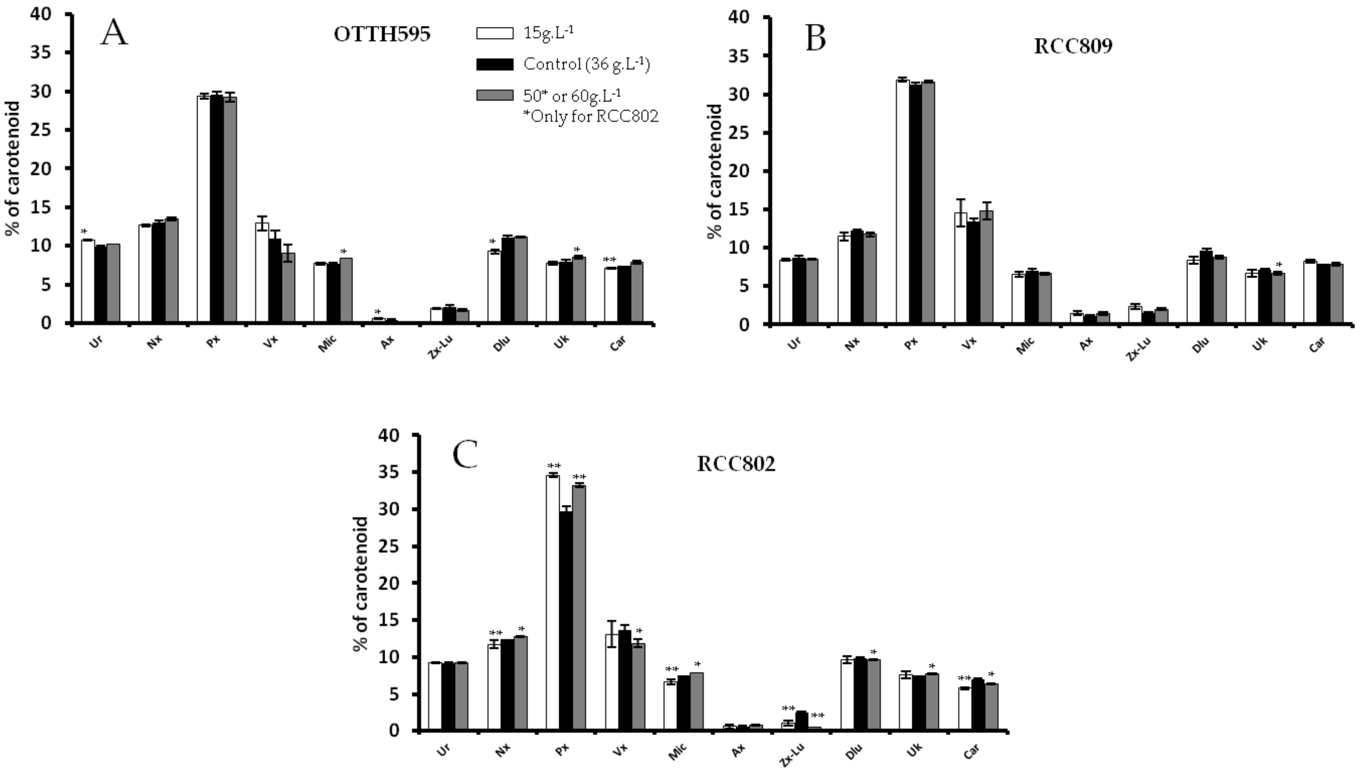

Figure S2 : Effect of salinity on the relative proportion of each carotenoid in *Ostreococcus* OTTH595 **(A**), RCC809 **(B)** and RCC802 **(C).** Cultures were exposed to salinity stress conditions of 15 $g.L^{-1}$ NaCl (white boxes), 36 $g.L^{-1}$ NaCl (black boxes and 50 or 60 $g.L^{-1}$ NaCl (grey boxes). Each carotenoid is expressed as percentage of the sum of uriolide (Ur), neoxanthin (Nx), prasinoxanthin (Px), violaxanthin (Vx), micromonal (Mic), antheraxanthin (Ax), zeaxanthin+lutein (Zx+Lu), dihydrolutein (Dlu), α+β carotene (Car)and one unknown carotenoid (Uk). Asterisks show significance in Student *T*-test(*: P < 0.05; **P < 0.01).

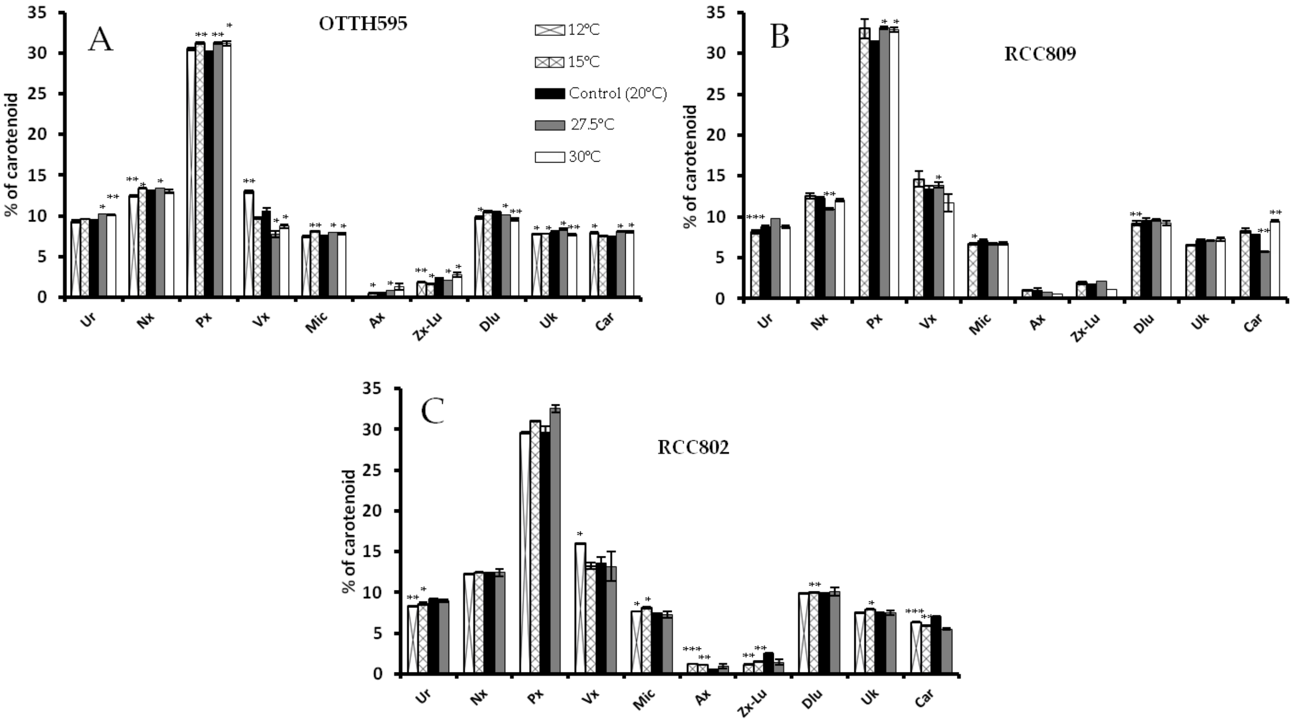

Figure S3: Effect of temperature on the relative proportion of each carotenoid in *Ostreococcus* OTTH595 (**A**),RCC809 **(B)** and RCC802 **(C).** Cultures were exposed to temperature conditions of 12°C, (large crosses) 15°C (small crosses), 20°C (control), 27.5°C (grey crosses) and 30°C(white boxes). Each carotenoid is expressed as percentage of the sum of uriolide (Ur), neoxanthin (Nx), prasinoxanthin (Px), violaxanthin (Vx), micromonal (Mic), antheraxanthin (Ax), zeaxanthin+lutein (Zx+Lu), dihydrolutein (Dlu), α+β carotene (Car)and one unknown carotenoid (Uk). Asterisks show significance in Student *T*-test (*: $P < 0.05$; **$P < 0.01$).

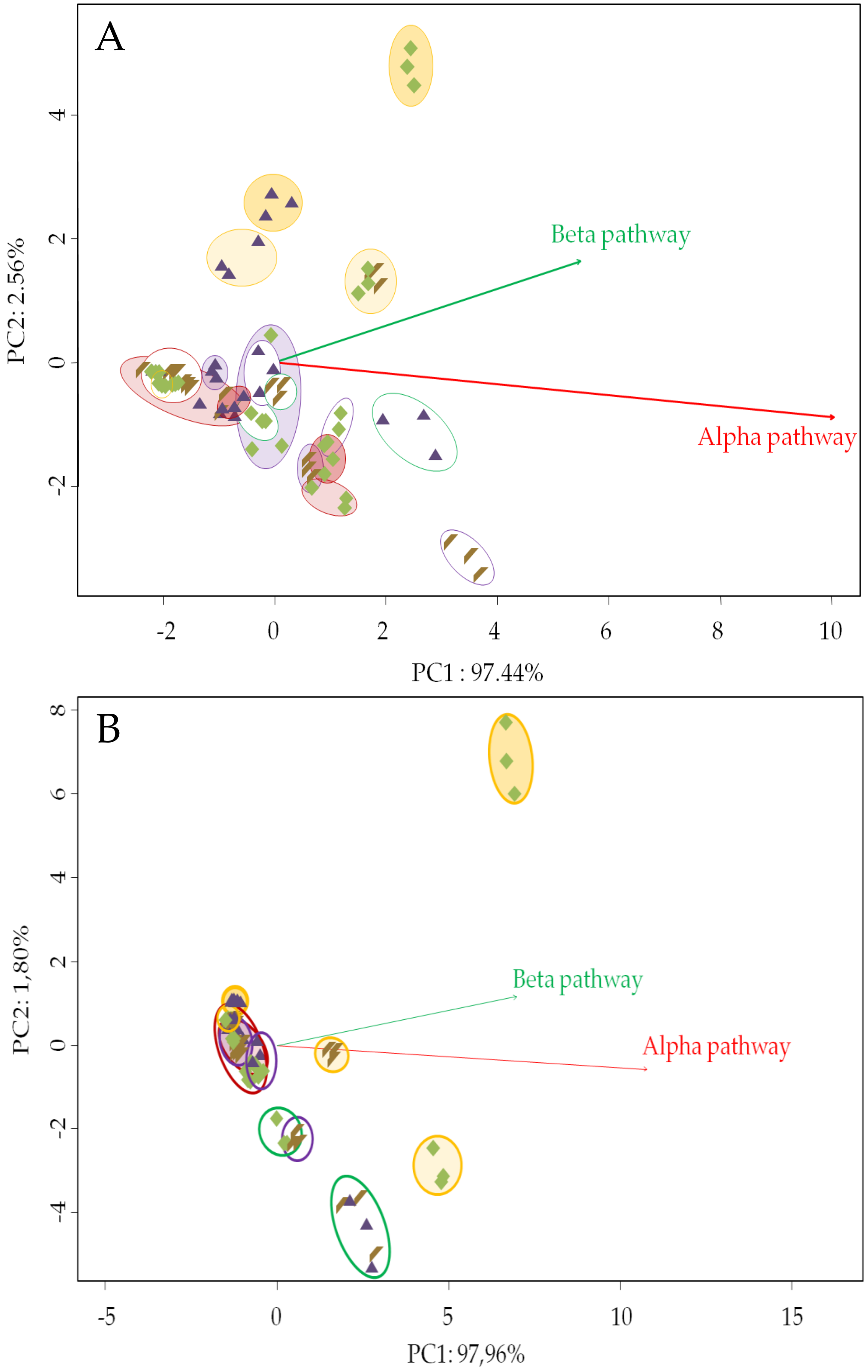

Figure S4: Redundancy analysis (RDA) of carotenoid batch productivity **(A)** and theoretical maximal productivity **(B)** in OTTH595 (green diamond), RCC809 **(**purple triangles) and RCC802 (brown trapezoid). **(C)** respectively. Red, purple, yellow correspond to temperature, salinity and light stresses conditions. Green is the standard control condition. Color intensity increases with the intensity of the applied stress. The length of each arrow represents the relative influence of carotenoid of the beta (red) or alpha (green) pathway on group separations .
